# Adaptation of Fatigue Affected Changes in Muscle EMG Frequency Characteristics for the Determination of Training Load in Physical Therapy for Cancer Patients

**DOI:** 10.1007/s12253-019-00668-3

**Published:** 2019-05-29

**Authors:** Adam Hegedus, Lukasz Trzaskoma, Peter Soldos, Kornelia Tuza, Peter Katona, Zsolt Greger, Fanni Zsarnoczky-Dulhazi, Bence Kopper

**Affiliations:** Department of Kinesiology, University of Physical Education, Budapest, Hungary

**Keywords:** Cancer treatment, physical rehabilitation, neuromuscular fatigue, Isometric, Motor unit activation

## Abstract

Cancer patients often experience loss in body weight and also a decrease in muscle mass, which results in the reduction of physical activity and mobilization of the patient. To decelerate the loss of muscle mass, as part of the cancer treatment patients frequently undergo physical therapy and considering the physical capabilities of the patients, with moderate loads. Moreover, frequent studies also observed for cancer patients, together with the decrease in muscle mass a shift into fast-twitch muscle fibers from slow-twitch fibers. The aim of our study therefore was to determine how motor fibers behave under moderate isometric load executed until total exhaustion. 11 university students (G1), and 14 elite athletes (G2) participated in the study. 65% of the maximal voluntary contraction (MVC) was determined for the biceps brachii muscle, and with this load holding a weight, participants had to sustain a 90 deg. isometric elbow flexion in a standing posture until complete fatigue occurred. EMG activity for the biceps brachii muscle was measured and frequency analysis was performed. 3 windows were determined in the fatiguing protocol: the first (W1), middle (W2), and last (W3) 5 s, and also frequency analysis for MVC was performed (MAX) between 0 and 260 Hz with 20 Hz wide frequency bands. The results indicate, that as the protocol progressed in time and the effect of fatigue increased (from W1 to W3) the activity of low frequency muscle fibers significantly increased (0-40 Hz) while activity of high frequency muscle fibers (60-260 Hz) significantly decreased for G1 and G2 groups identically. We can conclude, that training applied with constant moderate tension as fatigue increases will result in the increased activation of the lower frequency slow–twitch muscle fibers, but the increase of fatigue in the lower frequency fibers will not result in the increase in the activation level of the higher frequency fast-twitch fibers. Consequently, because as slow-twitch fibers are being used at moderate loads and even when fatigue occurs in these fibers the fast-twitch fibers will not work, higher muscle loads are needed if the aim is to activate fast-twitch fibers. Considering the shift into fast-twitch muscle fibers from slow-twitch fibers for cancer patients, in some cases if the patient’s age and physical status allows during the physical treatment, higher loads and consequently higher levels of activation might be beneficial for the retardment of loss concerning the fast-twitch fiber mass.

## Introduction

In cancer cachexia patients often face weight loss and also muscle atrophy, that results in the reduction of their quality of life, accelerates the progression of the cancer, results in worse surgical outcome and lower probability of survival [1–4].

Although loss of muscle mass can often be observed in cancer patients, our present understanding of the phenomenon is restricted. Some studies conclude, that muscle atrophy is a result of cancer, but also cancer treatment can be associated with the loss of muscle mass [5, 6]. To counteract muscle atrophy in patients, complex therapies are used including specialized nutrition supplementation, and also physical training [7, 8]. In physical training the different types of exercises result in different effect, for example endurance training stimulates the oxidative metabolisms, while does not result in significant muscle mass increase, while resistance training induces muscle hypertrophy [9, 10]. The problem with exercise programs applied for cancer patients, is that the status of the patients, for example cardiac dysfunction, chronic fatigue, etc. limits their exercise capacity. Therefore, to counteract muscle atrophy often resistance exercise protocols are used, but in most cases applied with moderate loads and consequently with moderate activation levels [11]. To induce muscle hypertrophy the defined magnitude of the activation and load is crucial, as moderate training load will only stimulate slow-twitch muscle fibers, but will not stimulate fast-twitch muscle fibers [12]. One more phenomenon that should be taken into account when definig training load in cancer patient physical treatment is that according to recent studies, in the case of cancer patients with the decrease in muscle mass simultaneously a shift into fast-twitch muscle fibers from slow-twitch fibers occur [13]. The decreased mass of the fatigue-resistant slow-twitch fibers are probably partially responsible for the exercise intolerance and rapid fatigue often observed in cancer patients. This means, that the specific training and activation of the fast-twitch fibers and the reduction of muscle atrophy has an extreme significance, when the aim is to sustain the patients’ general quality of life and mobility.

Consequently, as in many cases for cancer patients, when high intensity training is not applicable, the question may arise as to whether exercises applied with moderate load will activate the fast-twitch muscles, and as a result reduce muscle mass loss in these fibers. When applying resistance training with moderate loads the slow-twitch fibers will be active, but it can be speculated, that if these slow-twitch fibers fatigue but the training continues, the fast-twitch fibers might take over the role and will become active.

In the human neuromuscular system, the central nervous system regulates muscle force output by sending neural signals through alpha motoneurons to the muscles in two ways: by increasing the activation level if the signal, consequently recruiting and therefore activating more muscle fibers, and by altering the frequency of the stimuli [14]. Low frequency muscles are the type I, in other words slow-twitch muscle fibers. These fibers have a low rate of tension development, and low force output, but tend to resist fatigue. High frequency muscle fibers are the type IIa and IIx fast-twitch muscle fibers. The rate of tension development is high in these fibers, with a high force output, but these fibers tend to fatigue rapidly. The size principle is a significant factor in the regulation of force output [15, 16]. According to the size principle, as the force output increases more muscle fibers and simultaneously also higher frequency muscle fibers switch on [12, 17–22]. If the load is moderate, consequently the muscle activation is moderate, not all fibers are active. If the load is being kept at the same moderate level theoretically after the beginning of the contraction there would not be any visible significant change in muscle activation magnitude and pattern. But as muscle fatigue occur, it can be presumed, that while the neural activation remains constant not all muscle fibers will be able to maintain the contraction at the same level. Therefore, it can also be presumed, that – especially in isometric circumstances or in resistance exercises – the neural system would compensate to maintain the same level of contraction in the muscle that is required for further holding the moderate load, and consequently would elevate the activation level of the neural stimulus and also increase the frequency of the signal to activate new fibers.

**Fig. 1:**
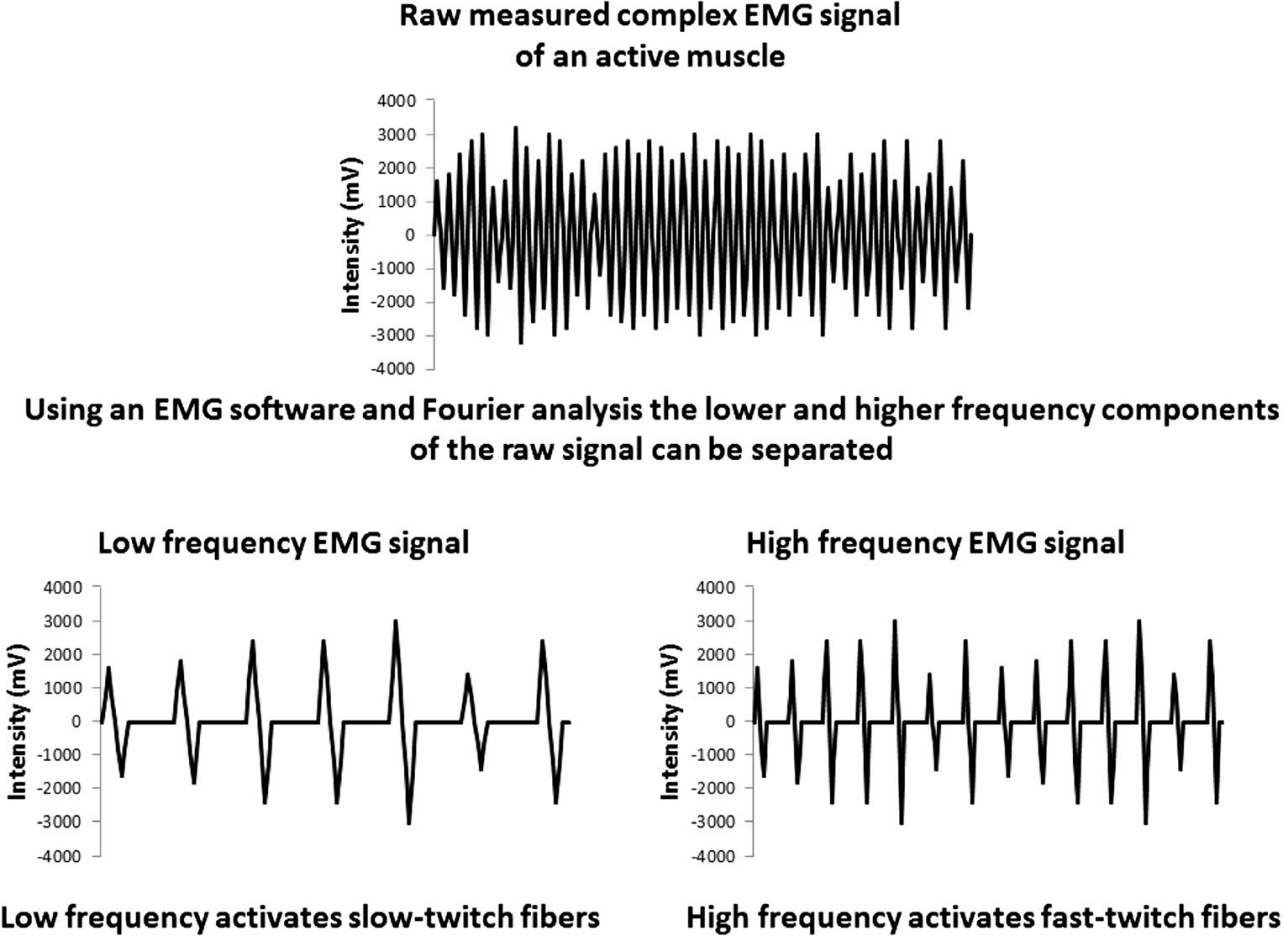
Raw measured EMG signal can be separated into lower and higher frequency components. As a transition from slow-twitch fibers to fast-twitch fibers can often be observed in cancer patients [13], to counteract loss of muscle mass, physical theraphy using higher loads that activates fast-twitch fibers through higher frequency signals can be beneficial

The aim of this study was to determine how the activation level of the biceps brahii muscle changes while the muscle reaches a completely fatigued state using moderate isometric load. To determine the activation level of different frequency muscle fibers, we have calculated the power spectrum content of the EMG data for 20 Hz wide frequency bands. The EMG signal, that can be recorded with surface electrodes, is a complex signal which consists of lower and higher frequency parts. Using the EMG software and Fourier analysis the lower and higher frequency components can be separated, and the amplitude, and consequently the intensity of the lower and higher frequency signals can be calculated, and as a result the activation level of the lower and higher frequency, slow and fast-twitch muscle fibers can be determined. (Figure 1). Using moderate (65% of maximal voluntary contraction-MVC) load it can be assumed, that at the beginning of the contraction the activity of the low frequency motor units would be high, while the activity of the high frequency motor units would be low. At the end of the protocol we hypothesize, that the activation of the high frequency muscle fibers would rise. The increase in the activity of the high frequency fibers would indicate that as less and less low frequency fibers take part in maintaining the stretch of the muscle as the result of complete fatigue, more and more high frequency fibers would take over the load. This would mean, that at the end of the protocol with using moderate load the high frequency fast-twitch muscles will also be activated. Consequently, in the physical rehabilitation of cancer patients using moderate load in resistance exercises fast-twitch muscle fibers could be activated. Therefore, our aim was to measure the activation frequency characteristics of the biceps brachii muscle in our subjects throughout a fatigue protocol, while establishing a constant moderate isometric tension in the muscle.

## Methods

15 male and 10 female subjects participated in the study. The subjects were the students of the University of Physical Education. The subjects were in good physical condition, prior to the study they did not report any upper limb injury. Age: 23,83 ± 5,02 yrs., body mass: 70,71 ± 7,99 kg, height: 176,91 ± 7,13 cm. The sample was divided into two groups: recreational university students without sports background (G1) and professional handball players (G2) As the fiber characteristics for the two groups is different it can also be hypothesized, that the activation pattern of the frequency distribution for the two groups during the protocol would be different. As G1 group presumably has a mixed fiber type distribution, and G2 has a fast-twitch dominant fiber distribution, with this construction of the study we can replicate the phenomenon, that in the case of cancer patients with the decrease in muscle mass simultaneously a shift into fast-twitch muscle fibers from slow-twitch fibers occur [13].

G1 consisted of 11 subjects (6 male, 5 female), age: 21,63 ± 1,6 yrs., body mass: 69,45 ± 6,78 kg, height: 174,81 ± 5,09 cm, and G2 consisted of 14 subjects (9 male, 5 female), age 25,57 ± 6,25 yrs., body mass 71,71 ± 10,74 kg, height 178,57 ± 9,33 cm, training age 13,71 ± 5,58 yrs. The subjects were informed about the study orally and signed a written agreement before participating in the study. The Ethical Committee of the University of Physical Education approved the protocol (Ethical approval number: TE-KEB/No06/2018), which was in union with the Declaration of Helsinki.

Surface EMG (sEMG) signals of the right biceps brachii muscle were recorded during the protocol. Before beginning the trial, the skin needed to be prepared to obtain better sEMG-recordings, to reduce the risk of imbalance between the electrodes, and noise. In order to achieve this, we removed the hair from the skin surface with a razor and the mortified epithelium with sandpaper, and finally washed the skin with alcohol [23]. Two unipolar Skintact F-55 electrodes were placed onto the muscle belly, between the tendon and the motor point in union with the SENIAM recommendation for the biceps brachii muscle, with an interelectrode distance of 4 cm and the ground electrode was placed onto the olecranon [24]. Noraxon MyoResearch Master Edition software was used for the detection of the sEMG signals, and for data processing.

**Fig. 2:**
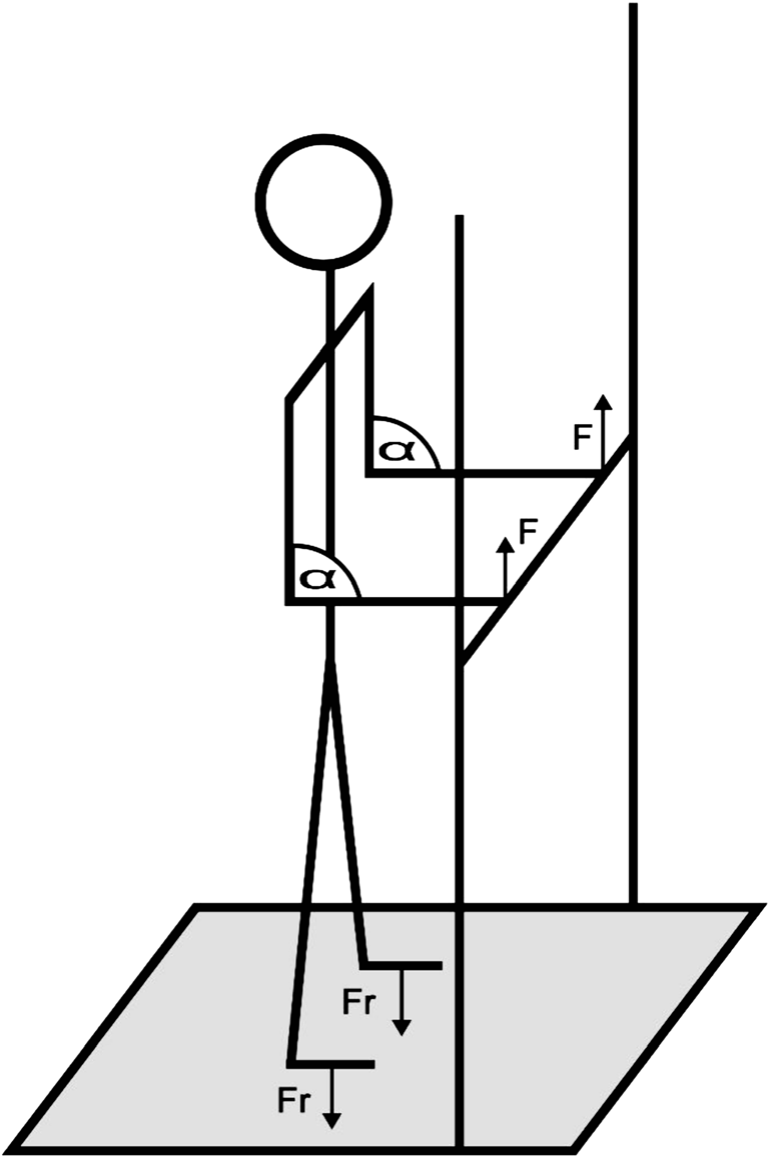
Subject standing on the force plateau. To determine the Maximal Voluntary Contraction of the elbow flexor muscles the subjects were standing on a force platform while holding a fixed rod. From the measured results the applied moderate load for the protocol could be calculated

Our aim was to study muscle activation under submaximal isometric load with the use of a controlled external load. To achieve this first the subjects’ maximal voluntary contraction (MVC) of the elbow flexor muscles were measured in 90 degrees elbow flexion. The subjects had their arms flexed α = 90 degrees in supinated position, while standing on a Kistler Force Platform System 92–81 B and holding a fixed rod (Fig. 2). Subjects were asked to gradually increase force output of the elbow flexor muscles, reaching their MVC. The results were recorded and from this data MVC 65% was calculated, which was the external load for the task. Olympic weightlifting rod and weightlifting weights were used for the external load. The subjects were asked to hold the rod at the 65% of MVC in 90 degrees elbow flexion while standing upright, until complete fatigue. After putting down the weight as the effect of complete fatigue, subjects were asked to do another MVC on the Kistler force plateau immediately (in less than 3 s) in the same way before. sEMG signals were recorded throughout the entire trial, and for the second MVC.

For processing sEMG data Noraxon MyoResearch Master Edition 1.08.27 software was used. Under 5 Hz low cut, over 350 Hz high cut, and 50–60 Hz frequency domains were filtered with the software. From the signals recorded throughout the trial three windows were cut: first 5 s (W1), middle 5 s (W2), and last 5 s (W3), and immediately after W3 signals for the maximal effort contraction was recorded (MAX), and also signals of the whole second MVC was processed. Between 0 and 260 Hz frequency spectrum intensity distribution was divided into 20 Hz frequency bands. Using the Noraxon software we have calculated the power spectrum content for the frequency analysis using Fourier transformation in the frequency bands.

The basic data was characterized by means and standard deviations. Because of the limited sample size and for the purpose of selecting the adequate statistical procedure Shapiro Wilk’s W test was calculated for checking the normal distribution of the data. The data was normally distributed, and for comparing the differences in different times for the same samples dependent T-test was used, for comparing the differences between the groups independent T-test was used. StatSoft STATISTICA 13.2 was used for the statistical analysis, Significance level was set for *p* < 0.05.

## Results

**Fig. 3:**
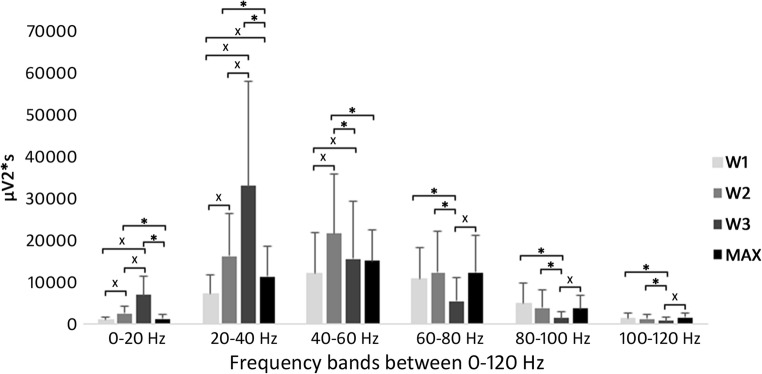
Motor unit activity during the protocol between 0 and 120 Hz (X indicates significant increase, * indicates significant decrease). From W1 to W3 for 0-60 Hz significant increase can be observed, while above 60 Hz significant decrease. While holding moderate load as the fatigue increases the activity of the slow-twitch fibers increases, while the activity of the fast-twitch fibres decreases. As Max (recorded instanly after the end of the protocol is significantly greater than W3 from 60 Hz suggests neural inhibition of the fast-twitch fibers

When comparing the data between the recreational university students (G1) and the professional handball players (G2) group, the G1 group had significantly greater activity in the W1 of 0–20 Hz frequency band, and MAX of 60–80 Hz frequency band, but we have not found significant difference for any other comparisons. As minimal difference could be observed in the EMG characteristics between the G1 and G2 groups, and taking into consideration the nature of EMG signals with relatively high standard deviations we have united the datasets for the two groups to calculate statistical difference between W1-W2-W3-Max to increase sample size (nG1 + G2 = 25 subjects), and consequently to increase the power of the statistical procedures and consequently the conclusions of the study. Comparing the results for the data of all of the participants significant increase (inc-increase, dec-decrease) was found in 0–20 Hz frequency band between W1-W2 (inc. 162%), W1-W3 (inc. 618%), W2-W3 (inc. 174%); 20–40 Hz frequency band between W1-W2 (inc. 120%), W1-W3 (inc. 347%), W1-MAX (inc. 55%), W2-W3 (inc. 103%); 40–60 Hz frequency band between W1-W2 (inc. 78%), W1-W3 (inc. 28%); 60–80 Hz frequency band between W3-MAX (inc. 120%); 80–100 Hz frequency band between W3-MAX (inc. 163%); 100–120 Hz frequency band between W3-MAX (inc. 90%); significant decrease was found in 0–20 Hz frequency band between W2-MAX (dec. 50%), W3-MAX (dec. 82%); 20–40 Hz frequency band between W2-MAX (dec. 30%), W3-MAX (dec. 66%); 40–60 Hz frequency band between W2-W3 (dec. 28%), W2-MAX (dec. 30%); 60–80 Hz frequency band between W1-W3 (dec. 48%), W2-W3 (dec. 55%); 80–100 Hz frequency band between W1-W3 (dec. 70%), W2-W3 (dec. 63%); 100–120 Hz frequency band between W1-W3 (dec. 41%), W2-W3 (dec. 35%) (Fig. 3).

**Fig. 4:**
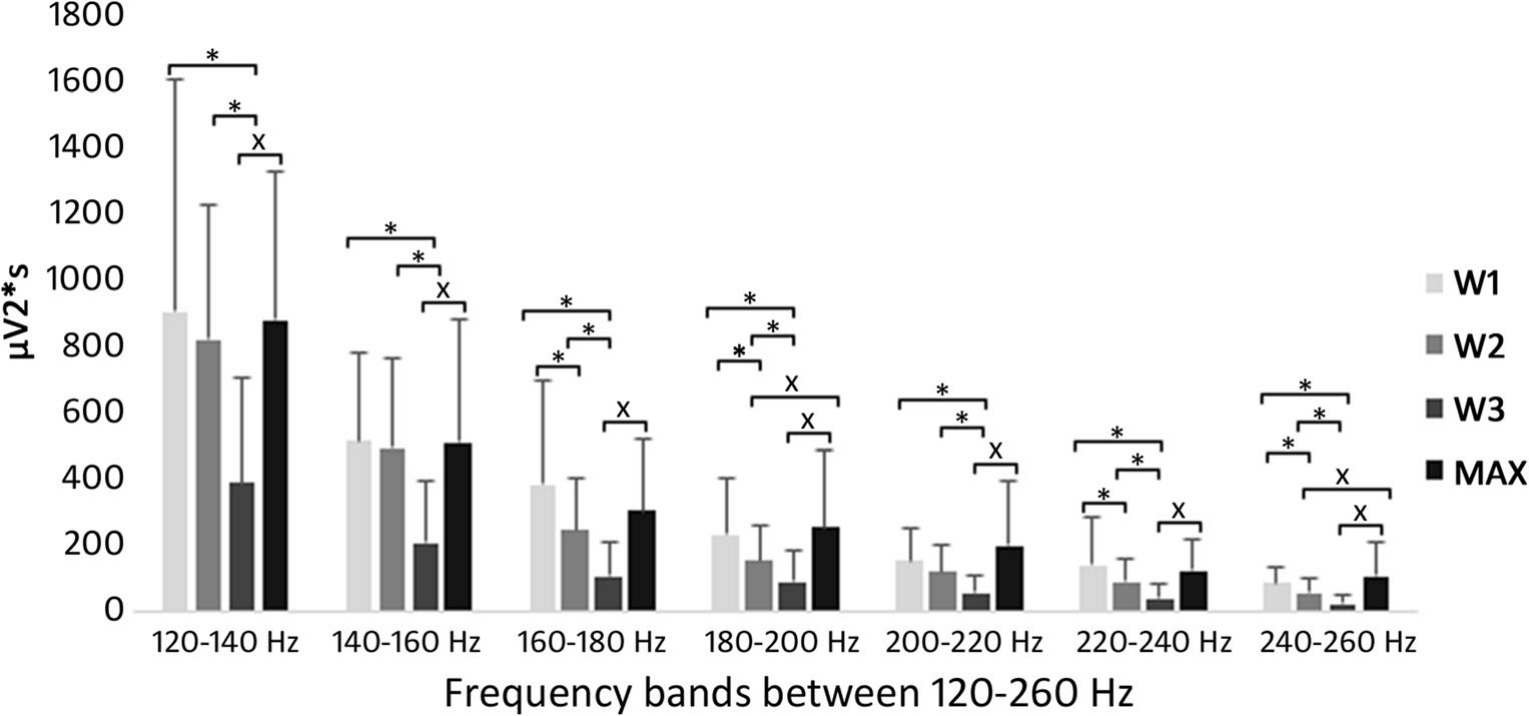
Motor unit activity during the protocol between 120 and 260 Hz (X indicates significant increase, * indicates significant decrease). From W1 to W3 above 120 Hz significant decrease can be observed in every comparisons. While holding moderate load as the fatigue increases the activity of the fast-twitch fibres decreases

Significant increase was found in 120–140 Hz frequency band between W3-MAX (inc. 124%); 140–160 Hz frequency band between W3-MAX (inc. 143%); 160–180 Hz frequency band between W3-MAX (inc. 183%); 180–200 Hz frequency band between W2-MAX (inc. 65%), W3-MAX (inc. 176%); 200–220 Hz frequency band between W3-MAX (inc. 237%); 220–240 Hz frequency band between W3-MAX (inc. 195%); 240–260 Hz frequency band between W2-MAX (inc. 81%), W3-MAX (inc. 304%); significant decrease was found in 120–140 Hz frequency band between W1-W3 (dec. 57%), W2-W3 (dec. 53%); 140–160 Hz frequency band between W1-W3 (dec. 59%), W2-W3 (dec. 58%); 160–180 Hz frequency band between W1-W2 (dec. 36%), W1-W3 (dec. 72%), W2-W3 (dec. 56%); 180–200 Hz frequency band between W1-W2 (dec. 34%), W1-W3 (dec. 61%), W2-W3 (dec. 41%); 200–220 Hz frequency band between W1-W3 (dec. 61%), W2-W3 (dec. 52%); 220–240 Hz frequency band between W1-W2 (dec. 35%), W1-W3 (dec. 70%), W2-W3 (dec. 54%); 240–260 Hz frequency band between W1-W2 (dec. 52%), W1-W3 (dec. 69%), W2-W3 (dec. 55%) (Fig. 4).

## Discussion

**Fig. 5:**
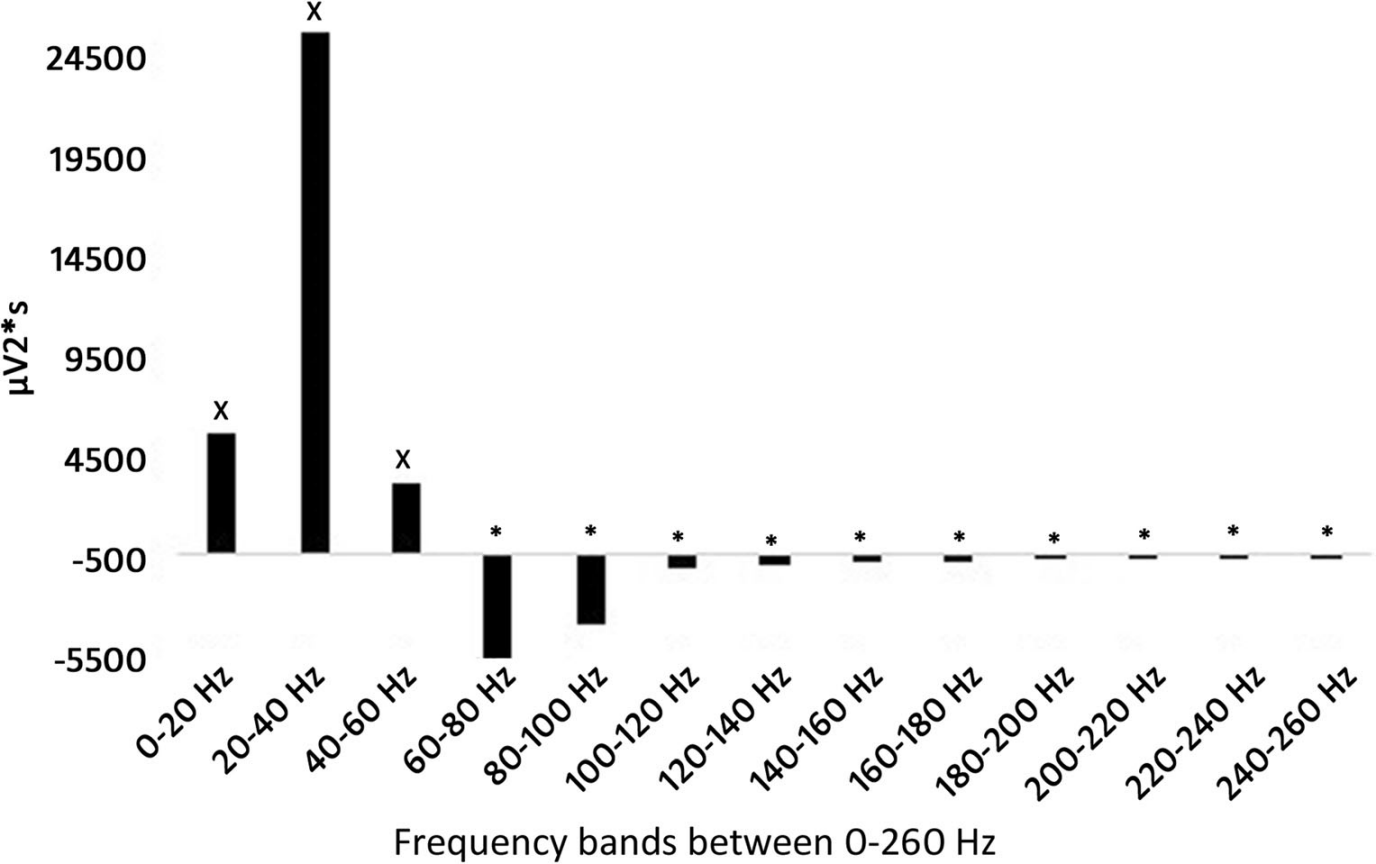
Tendency of frequency band changes from W1 to W3 (X indicates significant increase, * indicates significant decrease). From W1 to W3 for 0-60 Hz significant increase can be observed, while above 60 Hz significant decrease. The results suggest, that while applying moderate load as the fatigue in the slow-twitch fibers increase, the activation of the fast-twitch fibers will decrease

Our results indicate, that the activity of the low frequency muscle fibers will rise from the beginning of the contraction as the fatiguing protocol proceeds. But simultaneously the activity of the high frequency muscle fibers lowered from W1 to W3 as the fatiguing protocol proceeded (Fig. 5).

In case of the low frequency muscle fibers our results are correspond with previous studies [25, 26], as these studies show that the central nervous system tries to compensate fatigue with a higher level of muscle activation, and it is possible, that new slow-twitch fibers are recruited [12]. We hypothesized, that when the low frequency muscle fibers are fatigued, and the central nervous system cannot activate more low frequency fibers, the activity of high frequency fibers would increase, so these newly recruited fibers can disencumber the fatigued low frequency fibers. This phenomenon will expectedly occur at the end of our protocol, presumably at W3, where the activity of higher frequency muscle fibers consequently would rise. On the contrary our results indicate, that the activity of high frequency muscle fibers from 60 Hz reduced during the protocol. But interestingly the decrease of activity in the fast frequency fibers presumably did not occur due to their complete fatigue, because immediately after the fatigue protocol when we have recorded a voluntary maximal contraction at MAX much higher activity could be observed than at W3 for these fast-twitch fibers. These results indicate, that if the initial goal is to hold the weight as long as possible, as low frequency motor units are not able to maintain the tension in the muscle high frequency motor units will not take over the task. But interestingly the activation of the higher frequency motor units decrease as time passes by in the protocol, and even at complete fatigue would not increase.

In the case of cancer patients with the decrease in muscle mass simultaneously a shift into fast-twitch muscle fibers from slow-twitch fibers occur [13]. Therefore, the question may arise, that people who dominantly have more fast frequency motor units may have a different activation pattern and consequently the activation of low and high frequency motor units would behave differently in the protocol. This could be explained by some kind of neural difference for different motor unit distributions, or a better transition between low and high activation units. To investigate this problem besides recreational university students (G1), professional handball players (G2 - who would presumably have more fast frequency motor units in their upper extremity) were examined in our study, so therefore the two groups could be compared. We could not find significant difference in most of the cases in the comparisons in muscle activity pattern between the two groups consequently we have to conclude, that changes in muscle type distribution does not affect muscle activation behavior.

The phenomenon recorded as the decreased activation level of the fast-twitch fibers from W1 to W3 -as immediately after W3 at MAX the activation levels were significantly higher- would suggest some kind of neural inhibition from the central nervous system. We do not have a definite explanation for the recorded activation pattern of the fast-twitch fibers therefore for clearly explaining the phenomenon more studies are needed.

## Conclusion

Cancer patients often face cachexia and also in many cases muscle atrophy. To counteract muscle atrophy physical training is used, but considering the patients’ physical status predominantly with moderate load. Taking into consideration the phenomenon, that muscle fibers tend to switch from slow to fast twitch fibers in cancer patients we investigated the effect of moderate training load one slow and fast twitch fiber muscle activation. From our results we can conclude that moderate training load will not activate fast-twitch fibers – presumably because of neural inhibition-, consequently with moderate training load, it is not possible to counteract the atrophy of the fast-twitch fibers. Our results suggest that if the aim of the treatment is the activation of the fast-twitch fibers of cancer patients, and the age and physical status (according to ECOG performance status) of the individual is not a prohibiting factor, higher training load is beneficial for the retardment of loss in the fast-twitch fiber mass.
